# Transcriptome and metabolome analyses of cold and darkness-induced pellicle cysts of *Scrippsiella trochoidea*

**DOI:** 10.1186/s12864-021-07840-7

**Published:** 2021-07-10

**Authors:** Xin Guo, Zhaohui Wang, Lei Liu, Yang Li

**Affiliations:** 1 Guangzhou Key Laboratory of Subtropical Biodiversity and Biomonitoring, Guangdong Provincial Key Laboratory of Healthy and Safe Aquaculture, School of Life Science, South China Normal University, West 55 of Zhongshan Avenue, 510631 Guangzhou, China; 2 Department of Ecology, College of Life Science and Technology, Jinan University, West 601 of Huangpu Avenue, 510632 Guangzhou, China

**Keywords:** Dinoflagellates, Pellicle cysts, *Scrippsiella trochoidea*, Cold and darkness, Transcriptome, Metabolome

## Abstract

**Background:**

Dinoflagellates are a group of unicellular organisms that are a major component of aquatic eukaryotes and important contributors to marine primary production. Nevertheless, many dinoflagellates are considered harmful algal bloom (HAB) species due to their detrimental environmental and human health impacts. Cyst formation is widely perceived as an adaptive strategy of cyst-forming dinoflagellates in response to adverse environmental conditions. Dinoflagellate cysts play critical roles in bloom dynamics. However, our insight into the underlying molecular basis of encystment is still limited. To investigate the molecular processes regulating encystment in dinoflagellates, transcriptome and metabolome investigations were performed on cold and darkness-induced pellicle cysts of *Scrippsiella trochoidea*.

**Results:**

No significant transcriptional response was observed at 2 h; however, massive transcriptome and metabolome reprogramming occurred at 5 h and in pellicle cysts. The gene-to-metabolite network demonstrated that the initial transformation from vegetative cells into pellicle cysts was highly energy demanding through the activation of catabolism, including glycolysis, β-oxidation, TCA cycle and oxidative phosphorylation, to cope with cold-darkness-induced stress. However, after transformation into pellicle cysts, the metabolism was greatly reduced, and various sugars, polyunsaturated fatty acids and amino acids accumulated to prolong survival. The identification of 56 differentially expressed genes (DEGs) related to signal transduction indicated that *S. trochoidea* received a cold-darkness signal that activated multiple signal transduction pathways, leading to encystment. The elevated expression of genes encoding enzymes involved in ROS stress suggested that pellicle cysts respond to increased oxidative stress. Several cell cycle-related genes were repressed. Intriguingly, 11 DEGs associated with sexual reproduction suggested that pellicle cysts (or some portion thereof) may be a product of sexual reproduction.

**Conclusions:**

This study provides the first transcriptome and metabolome analyses conducted during the encystment of *S. trochoidea*, an event that requires complex regulatory mechanisms and impacts on population dynamics. The results reveal comprehensive molecular regulatory processes underlying life cycle regulation in dinoflagellates involving signal transduction, gene expression and metabolite profile, which will improve our ability to understand and monitor dinoflagellate blooms.

**Supplementary Information:**

The online version contains supplementary material available at 10.1186/s12864-021-07840-7.

## Background

Dinoflagellates are a phylum of unicellular, generally marine eukaryotes that are most closely related to ciliates and apicomplexans [1]. As one of the major groups of marine phytoplankton, they are important contributors to global primary production [2]. Nevertheless, many dinoflagellate species are also a rich source of marine toxins and can form harmful algal blooms (HABs) [3]. HABs have received tremendous scrutiny due to their detrimental environmental and human health impacts [4]. The formation of HABs involves the intersection of physical, chemical and biological processes that are often specific to HAB species [5, 6].

More than 10 % of the approximately 2000 known marine dinoflagellate species produce cysts within their life cycle [7]. Encystment is one of the adaptive strategies of dinoflagellates and allows them to alternately inhabit the benthos during unfavorable environmental conditions and the water column when favorable conditions are restored [7, 8]. Thus, cysts play critical roles in the ecology of dinoflagellates, particularly the dynamics of HABs, as encystment can decrease the density of vegetative cells in the water column and cysts can act as a reservoir for new populations [7–9]. Normal viable dinoflagellate cells have been shown to germinate from cysts found in century-old sediments [10]. Dinoflagellates can form two basic types of cysts, resting cysts and pellicle cysts [11]. Generally, resting cysts are produced by sexual reproduction, have a thick cyst wall and exhibit a mandatory dormancy period [12], whereas pellicle cysts are asexual or sexual origin, have a thin cyst wall and usually do not require a maturation period [13].

To our knowledge, at least 48 dinoflagellate species have been reported to form pellicle cysts under both laboratory and natural conditions [14]. Pellicle cysts form readily under various environmental stresses, such as nutrient stress [12], mechanical shock [15], changes in temperature [16], darkness [17], parasite infection [18] and even passage through the digestive tract of oysters [19]. Intriguingly, melatonin treatment or changes in photoperiod induce pellicle cyst formation, providing an intriguing connection to the biological clock [20]. Furthermore, several *in situ* studies have shown that there is a cycle of pellicle cyst formation, possibly related to light cycles, in which encystment and excystment appear to be controlled by light [21, 22]. Thus, although pellicle cysts are common among dinoflagellate species, the factors triggering the formation of pellicle cysts from motile cells (and vice-versa) are unclear. The metabolism of dinoflagellate cysts is dramatically reduced to allow them to resist poor environmental conditions and survive for an extended period in dormancy [16, 23]. Roy et al. [16] proposed that the highly reduced metabolism in cysts is achieved by alterations in the levels of protein phosphorylation.

The thecate dinoflagellate *Scrippsiella trochoidea* is one of the causative species of nearshore blooms found worldwide and is also a common dominant dinoflagellate in China [6, 24]. Blooms of this species have been reported in Japan, Korea, USA, Europe and China [6, 25, 26], which can exhibit a high cell density and lead to hypoxia, resulting in fish kills [27]. Although *S*. *trochoidea* is nontoxic, recent research has shown that it can cause lethal effects on shellfish larvae [28]. In addition, *S. trochoidea* is recognized as being successfully adapted to a broad range of temperatures (5–30 °C), the optimal temperature for growth is 25 °C [29, 30]. Furthermore, the growth rate and biomass increased with the extension of photo phase in a certain range (9–14 h) [31]. Laboratory researches have shown that both low temperature and low light conditions induce pellicle cyst formation [32, 33]. More importantly, *S. trochoidea* is well known for its ability to easily form pellicle cysts in the field, and encystment and excystment play pivotal roles in the occurrence and collapse of its blooms [8]. Indeed, several lines of evidence have demonstrated that multiple adaptive strategies such as broad temperature tolerance and cyst formation contribute to the ecological success of *S. trochoidea* [34, 35].

Although encystment is an intricate process, advanced genetic analysis tools have made it possible to elucidate the complex process involved in pellicle cyst formation. In this study, we integrated transcriptome and metabolome analyses of pellicle cysts formed by *S. trochoidea* in response to low temperature and darkness to understand the molecular basis of the profound cellular changes resulting in encystment. Our results provide important insights into the gene expression and metabolite profile of pellicle cysts, which will provide a strong basis for the further characterization of key regulatory genes and metabolites involved in the processes of encystment and excystment.

## Results

### Cold and darkness induce pellicle cysts in ***Scrippsiella trochoidea***

**Fig. 1:**
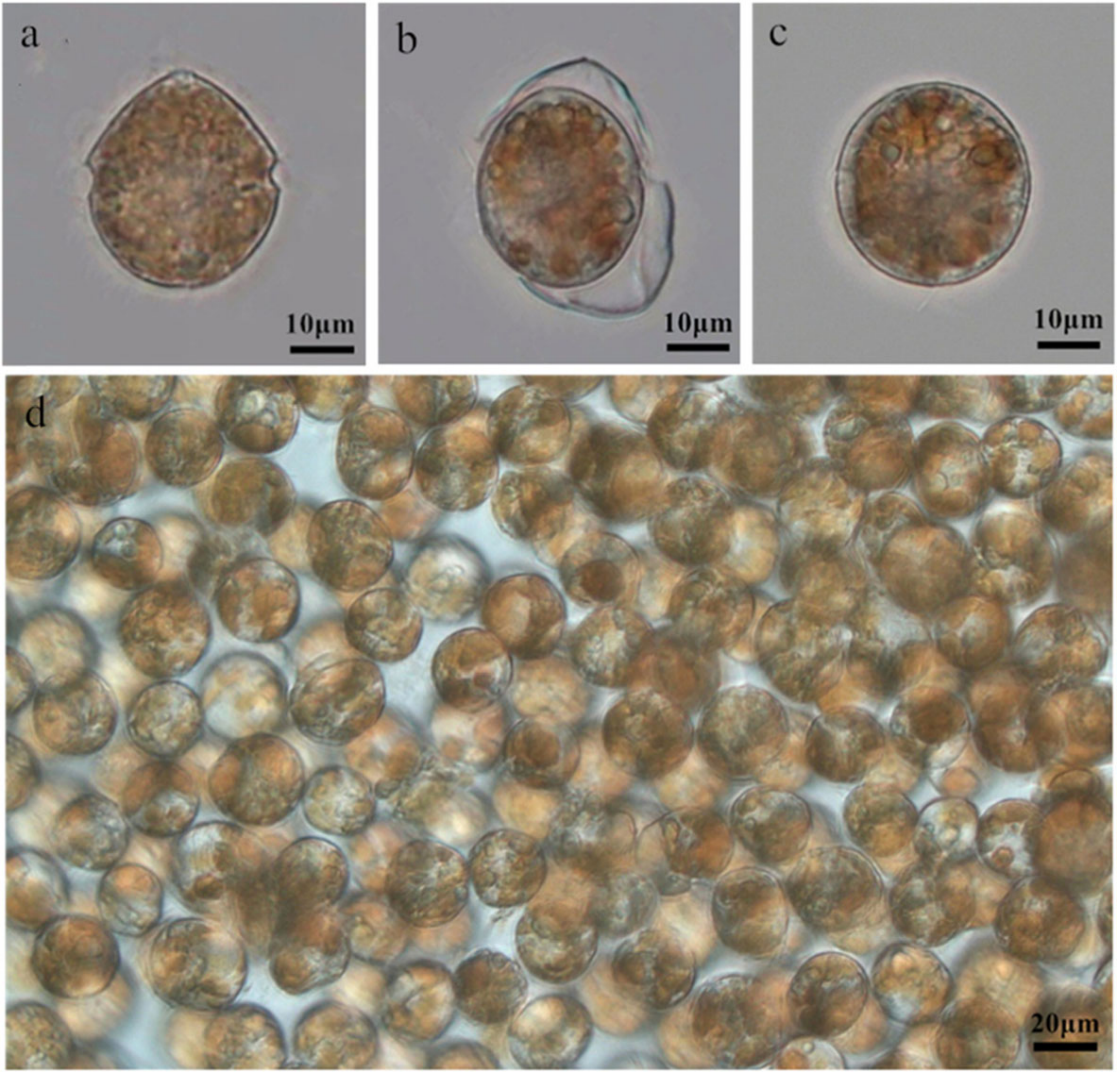
Light microscopy images of the vegetative cell and pellicle cysts of *Scrippsiella trochoidea*. (**a**) vegetative cell; (**b**) cyst formation by ecdysis; (**c**) pellicle cyst; (**d**) pellicle cysts collected by centrifugation

Within 2 h of treatment, a small proportion of vegetative cells lost swimming ability. At 5 h, approximately 7.5 % of cells became non-motile and settled at the bottom of the flask, and these cells shed their flagella as well as their theca (Fig. 1b). At day 3, the cells were round in shape and had a thin cell wall, consistent with the formation of pellicle cysts in other dinoflagellates (Fig. 1c), and the cyst formation rate was 15.9 % [33]. The diameter of the *S. trochoidea* pellicle cysts (21–32 μm) was similar to that of the vegetative cells (Fig. 1a). After centrifugation with Percoll, intact pellicle cysts could be collected (Fig. 1d). The pellicle cysts transformed back into motile cells within 24 h after exposure to normal growth conditions, the germination rate was 22.2 % [33]. We did not observe any mandatory dormancy period, indicating that the transition was relatively rapid. Taken together, we further investigated the gene expression patterns of *S. trochoidea* under cold and darkness at 2 h (D2), 5 h (D5) and in pellicle cysts (PC) compared with vegetative cells (CK).

### Transcriptome sequencing and functional annotation

A total of 321,920,359 clean reads were obtained from 12 samples, corresponding to 95.98 Gb of clean data, and the details of the transcriptome sequencing data are shown in supplementary Table S1. The probability of incorrect base calling was used to evaluate the sequencing quality according to the Q30 value, where a high ratio of values ≥ Q30 (> 92 %) for each sample indicated high RNA-seq quality. The high quality clean reads were *de novo* assembled into 100,132 unigenes with an average length of 1,087 bp and an N50 length of 1,740 bp (Table 1). Finally, we mapped the clean reads to the assembled unigenes, and over 75 % of the sequences in each sample were matched, indicating that the set of assembled unigenes was appropriate for differential expression analysis (supplementary Table S1).

Because of the absence of genomic information for *S. trochoidea*, to acquire the most informative and complete annotation, the assembled unigenes were subjected to BLAST searches against public databases, including Nr, GO, COG, KOG, eggNOG, KEGG, Swiss-Prot and Pfam. A total of 46,512 unigenes (46 % of the total) generated successful BLAST hits against known sequences or putative functions in at least one of the aforementioned public databases (supplementary Table S2). In the Nr species distribution, most of the unigene hits were for *Symbiodinium microadriaticum* (29,165, 67.46 %), *Emiliania huxleyi* (1,323, 3.06 %) and *Chrysochromulina* sp. (964, 2.23 %) (supplementary Fig. S1). Through alignment with the GO database, 8,604 unigenes were assigned to 46 GO classification terms (supplementary Fig. S2). Furthermore, 18,301 unigenes were annotated in the KOG database and grouped into 25 KOG categories (supplementary Fig. S3). The largest cluster was “general function prediction only”, followed by “signal transduction mechanisms” and “posttranslational modification, protein turnover, chaperones”. In addition, a total of 11,364 unigenes were assigned to 125 KEGG pathways, and the top 5 pathways were ribosome (374 unigenes), protein processing in endoplasmic reticulum (372), carbon metabolism (343), plant-pathogen interaction (342), biosynthesis of amino acids (323). Moreover, a total of 15,079 and 31,883 unigenes were annotated to Swiss-Prot and Pfam databases, respectively.

**Table 1:** Summary of RNA-seq assembly for *Scrippsiella trochoidea*

Category	Unigene
Total length (bp)	108,800,666
Total number	100,132
N50 (bp)	1740
GC %	61.97
Mean length (bp)	1087

### Global changes in gene expression during encystment

**Fig. 2:**
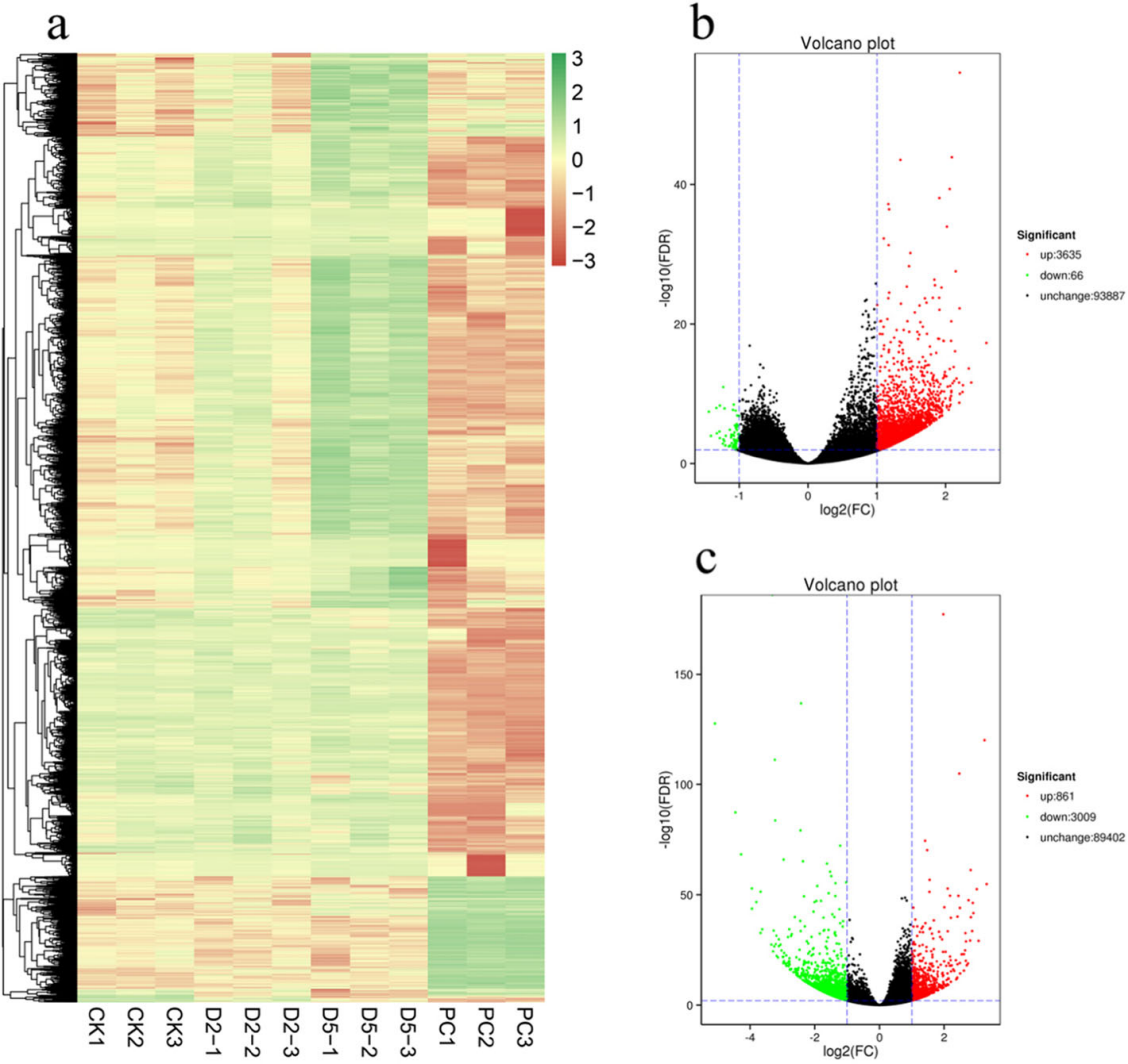
Global gene expression profiles and differentially expressed genes (DEGs) during the formation of pellicle cysts. (**a**) Hierarchical cluster analysis (HCA) of DEGs in the CK, D2, D5 and PC (3 replicates for each sample); (**b**) Volcano plot of gene expression levels comparing CK to D5 showing the number of DEGs in red (up-regulated) and green (down-regulated), and those that are unchanged are in black; (**c**) Volcano plot of gene expression levels comparing CK to PC. The color scale represents the log_2_-transformed FPKM values. CK represents the control group of vegetative cells in the exponential phase; D2 and D5 represent the groups treated with cold and darkness for two hours and five hours, respectively; PC represents the group of pellicle cysts

To analyse the differences and similarities among transcriptomes, hierarchical cluster analysis (HCA) was conducted to reveal the expression pattern of genes in three replicates in the CK, D2, D5 and PC groups. The results demonstrated distinct differences in gene expression profiles between PC and CK and between D5 and CK, in contrast to the highly similar expression profiles between D2 and CK (Fig. 2a). Since only two genes showed expression differences in D2, we further limited our focus to the differences between D5 or PC and CK. In the comparison of CK vs. D5, 3,701 DEGs (3,635 up-regulated and 66 down-regulated) were identified (Fig. 2b). In the comparison of CK vs. PC, 3,870 DEGs (861 up-regulated and 3,009 down-regulated) were found (Fig. 2c). A total of 570 and 1,505 DEGs were functionally annotated in D5 and PC, respectively (supplementary Table S3).

The DEGs were further subjected to GO enrichment analysis (supplementary Table S4). Table 2 shows the top 5 enriched GO terms of the DEGs. In the comparisons of CK and D5, we found that the most strikingly enriched GO terms (*p* < 0.01) were linked to translation, oxidation-reduction process, and ATP hydrolysis coupled proton transport in the category of biological process. In the molecular function category, structural constituent of ribosome, chlorophyll binding and cation transporting ATPase activity were the most significantly enriched terms, while terms associated with ribosomes were remarkably enriched in the cellular component category. In the comparisons of CK and PC, the most significantly enriched GO terms were related to ATP metabolism, photosynthesis, oxidation-reduction process, translation, cation/proton transporting ATPase and ribosome.

Additionally, the KEGG pathway classification provided deeper insights into the metabolic pathways related to the formation of pellicle cysts. All DEGs were mapped to the KEGG database, and 191 and 139 DEGs were annotated and assigned to 69 and 70 metabolic pathways based on the comparisons of CK vs. D5 and CK vs. PC, respectively (supplementary Table S5). Accordingly, several significantly enriched (*p* < 0.01) pathways were observed. In D5, the most notably enriched pathways were ribosome (ko03010), proteasome (ko03050) and oxidative phosphorylation (ko00190). In PC, however, protein processing in endoplasmic reticulum (ko04141), riboflavin metabolism (ko00740), photosynthesis (ko00195) and valine, leucine and isoleucine degradation (ko00280) were significantly enriched (supplementary Table S5).

**Table 2:** Top 5 enriched GO terms of the differentially expressed genes during encystment

	GO ID	Term	Annotated	Significant	Expected	KS
CK vs. D5						
BP	GO:0006412	translation	482	99	17.58	2.50E-14
	GO:0055114	oxidation-reduction process	367	23	13.39	8.40E-06
	GO:0015991	ATP hydrolysis coupled proton transport	32	3	1.17	2.40E-05
	GO:0009765	photosynthesis, light harvesting	50	2	1.82	1.70E-04
	GO:0006108	malate metabolic process	11	1	0.4	4.60E-04
MF	GO:0003735	structural constituent of ribosome	299	92	10.62	3.00E-22
	GO:0016168	chlorophyll binding	47	1	1.67	6.70E-04
	GO:0019829	cation transporting ATPase activity	45	2	1.6	7.20E-04
	GO:0030060	L-malate dehydrogenase activity	8	1	0.28	8.00E-04
	GO:0009055	electron carrier activity	45	9	1.6	8.60E-04
CC	GO:0022627	cytosolic small ribosomal subunit	73	29	3.47	1.10E-09
	GO:0022625	cytosolic large ribosomal subunit	111	36	5.28	2.50E-08
	GO:0005840	ribosome	337	97	16.02	1.90E-05
	GO:0009535	chloroplast thylakoid membrane	76	3	3.61	2.20E-03
	GO:0015935	small ribosomal subunit	93	34	4.42	4.00E-03
CK vs. PC						
BP	GO:0015991	ATP hydrolysis coupled proton transport	32	4	0.94	3.20E-06
	GO:0009765	photosynthesis, light harvesting	50	3	1.48	2.90E-05
	GO:0018298	protein-chromophore linkage	46	3	1.36	6.90E-05
	GO:0055114	oxidation-reduction process	367	12	10.83	9.20E-05
	GO:0006006	glucose metabolic process	47	2	1.39	1.09E-03
MF	GO:0016168	chlorophyll binding	47	3	1.52	4.90E-05
	GO:0016616	oxidoreductase activity, NAD or NADP as acceptor	66	1	2.14	6.20E-04
	GO:0019829	cation transporting ATPase activity	45	1	1.46	6.20E-04
	GO:0003677	DNA binding	87	9	2.82	6.40E-04
	GO:0003735	structural constituent of ribosome	299	17	9.69	8.50E-04
CC	GO:0009535	chloroplast thylakoid membrane	76	4	2.3	3.20E-04
	GO:0034357	photosynthetic membrane	97	8	2.94	3.20E-04
	GO:0005840	ribosome	337	17	10.22	5.40E-04
	GO:0044454	nuclear chromosome part	32	1	0.97	3.98E-03
	GO:0045263	proton-transporting ATP synthase complex, coupling factor F_0_	8	3	0.24	4.13E-03

CK represents the control group of vegetative cells in the exponential phase, D5 represents the group treated with cold and darkness for five hours, PC represents the group of pellicle cysts. BP, MF and CC represent biological process, molecular function and cellular component, respectively.

### Signal transduction

We searched the DEGs involved in cellular signal transduction, as the perception of external cues inevitably triggers signal transduction cascades. We identified 10 DEGs responsible for calcium transport that were up-regulated by 2.0-4.7-fold during the formation of pellicle cysts. In addition, 3 DEGs encoding calmodulin (CaM), CBL-interacting protein kinase (CBL) and calcium-dependent protein kinase (CDPK) were up-regulated by 2.0-3.1-fold at 5 h. In pellicle cysts, a total of 23 genes encoding CaMs, CBLs and CDPK family proteins were differentially expressed, among which 13 genes were up-regulated and 10 genes were down-regulated (supplementary Table S6).

Eight genes involved in phospholipid signaling were differentially expressed, almost all of which (6 genes) were up-regulated (supplementary Table S6). These genes encoding two G protein subunits, two GTP-binding proteins, one phospholipase C2-like protein, and three phosphatidylinositol 4-phosphate 5-kinases (PIP5Ks). Because all of these genes function in phospholipid-based signaling, their induction/repression by cold and darkness strongly suggests that phospholipid second messengers are an important component of signaling during encystment. Additionally, mitogen-activated protein kinase (MAPK) cascades organized by three-tiered modules (MAPKKK-MAPKK-MAPK) are considered to be involved in abiotic stress signaling. We identified 8 up-regulated genes related to MAPK cascades, including ras-domain containing protein, receptor-like kinase (RLK), MAPKKK3, MAPKK2 and MAPK related protein kinases (supplementary Table S6).

### Energy metabolism

A total of 63 DEGs were shown to be relevant to energy metabolism during the formation of pellicle cysts in *S. trochoidea*, which belonged to glycolysis, citrate cycle (TCA cycle), oxidative phosphorylation and fatty acid metabolism (supplementary Table S7). Elevated expression of 11 DEGs related to glycan metabolism, including cell wall alpha-1,3-glucan synthase, alpha-glucosidase, beta-glucosidase, endoglucanase, alpha-amylase, glucan-1,3-beta-glucosidase and cellulose, was observed during the formation of pellicle cysts. Additionally, 7 DEGs encoding enzymes involved in glycolysis and TCA cycle were up-regulated by 2.0-3.6-fold at 5 h, including glucose-6-phosphate isomerase, pyruvate dehydrogenase E1 component, dihydrolipoamide dehydrogenase, citrate synthase, succinyl-CoA synthetase and malate dehydrogenase. In pellicle cysts, DEGs (8 out of 9 DEGs) associated with glycolysis and TCA cycle were down-regulated by 2.1-3.8-fold. Among the differentially regulated oxidative phosphorylation genes, 12 DEGs encoding components of NADH dehydrogenase, cytochrome c reductase, cytochrome c oxidase, cytochrome c1, electron transfer flavoprotein subunit beta, ATP synthase subunit delta and ATP synthase subunit beta were up-regulated at 5 h, while oxidative phosphorylation associated genes encoding flavodoxin and ATP synthase subunit gamma were down-regulated in pellicle cysts. In addition, two genes encoding adenosine kinase (ADK), which plays a crucial role in regulating the equilibrium between ADP and ATP levels, presented ~ 2.4-fold up-regulation at 5 h, and one of these genes was down-regulated by 2.9-fold in pellicle cysts. Finally, 19 DEGs encoding enzymes for fatty acid metabolism, including acyl-CoA dehydrogenase, enoyl-CoA hydratase, hydroxyacyl-CoA dehydrogenase, acetyl-CoA acetyltransferase (thiolase), propionyl-CoA carboxylase, acetyl-CoA carboxylase, long-chain 3-oxoacyl-CoA reductase, fatty acid desaturase and fatty acid elongase, were differentially expressed.

### Stress response

We found 53 DEGs to be responsive to stress during encystment (supplementary Table S8). Among the 53 DEGs, approximately 50 % were identified as encoding heat shock proteins (HSPs), chaperones and DnaJ protein homologues. In addition to the large number of HSPs and chaperones genes, 16 DEGs involved in DNA replication and repair as well as chromatin assembly, exhibited reduced expression in pellicle cysts. Genes encoding enzymes related to the classic reactive oxygen species (ROS) detoxification pathway, such as ascorbate peroxidase (*APX*) and glutaredoxin (*Grx*), were induced by 2.1-fold and 2.3-fold, respectively, at 5 h. However, thioredoxins (*Trxs*, 2 DEGs), catalase (*CAT*, 1 DEG), glutathione S-transferases (*GSTs*, 2 DEGs), and *APX* (1 DEG) were induced by 2.1-7.4-fold in pellicle cysts. Additionally, 2 DEGs encoding cytochrome P450 (CYP450) were repressed by more than 2.5-fold. We also observed 2 DEGs encoding cold shock domain-containing proteins (CSPs) that were up-regulated by ~ 2.6-fold at 5 h.

### Cell cycle

Since dinoflagellate cysts are specialized cells with suspended cell division, genes related to cell cycle may be linked to encystment. According to our data, 6 DEGs were annotated as cyclins, cyclin-dependent kinases (CDKs) and cell division cycle protein with fundamental roles in the control of cell cycle, which were suppressed by more than 2.3-fold in pellicle cysts. Intriguingly, 11 genes potentially involved in sexual reproduction displayed differential expression between vegetative cells and pellicle cysts. Among them, 10 DEGs were homologues of the *MEI2*-like genes, 1 DEG was annotated as structural maintenance of chromosomes protein 3 (*SMC3*) (supplementary Table S9).

### Metabolome profiling

Given the highly similar expression profiles between D2 and CK, the 5 h treatment samples and pellicle cysts were collected and subjected to metabolome analysis using UHPLC-QTOF-MS. Orthogonal projections to latent structures-discriminant analysis (OPLS-DA) revealed that metabolic features formed distinct clusters (6 samples in each group) in the comparisons of CK vs. D5 and CK vs. PC in both positive and negative ion modes. Our results (Q2Y > 0.5) indicated that this model provided good predictability without overfitting, and this difference was highly significant (*p* = 5 × 10^− 3^, permutation test) (supplementary Fig. S4). A total of 80 and 987 molecular features showed differential accumulation in D5 and PC, and we tentatively labeled 17 and 111 species, respectively (supplementary Table S10). These differentially accumulated metabolites represented distinct metabolic categories based on their KEGG classifications. In our data set, large amounts of sugars, fatty acids, amino acids and organic acids were annotated to primary metabolic pathways involving glycolysis, TCA cycle, lipid metabolism and amino acid metabolism.

At the level of individual metabolites, cold and darkness induced changes in the relative abundance of multiple compounds. Several differentially accumulated metabolites were observed at 5 h; in particular, relative decrease in oleic acid concentrations (0.3-fold) with concomitant increases in the levels of glucose (2.2-fold), fructose (2.0-fold), betaine (2.1-fold), arginine (2.0-fold), glycine (2.2-fold), glycerol 3-phosphate (2.3-fold) and phosphocreatine (3.0-fold) were observed. However, the greatest changes were observed in pellicle cysts and were associated with high accumulation of saccharides, such as glucose (3.8-fold), fructose (5.2-fold) and trehalose (5.8-fold); amino acids, including proline (2.0-fold), alanine (2.8-fold), phenylalanine (4.7-fold), arginine (5.2-fold), glutamate (5.8-fold), glutamine (22.9-fold), glycine (8.2-fold), threonine (4.0-fold) and the nonprotein amino acid γ-aminobutyric acid (GABA, 4.8-fold); and a variety of polyunsaturated fatty acids (PUFAs), such as linolenic acid (3.1-fold), arachidonic acid (3.3-fold), palmitoleic acid (2.2-fold) and eicosadienoic acid (2.0-fold) (supplementary Table S10).

## Discussion

### General transcriptome features and global changes in gene expression during encystment

Our *de novo* transcriptome assembly obtained 100,132 non-redundant unigenes, a number consistent with previous studies that have reported ~ 49–118 K transcripts in different dinoflagellates [36–39]. Approximately half of the unigenes could be assigned annotations via comparison with available databases, a number similar to that for the most recently published transcriptome of *S. trochoidea* [40] and those obtained for other dinoflagellates via RNA-seq [38, 39, 41]. The limited functional annotation of genes in dinoflagellates was not surprising, as it is at least partly due to the paucity of available genome sequences and highly limited studies on functional genes [39, 40].

Transcriptional patterns were highly similar between D2 and CK, with only two up-regulated DEGs being identified at 2 h; one of these DEGs was functionally annotated as casein kinase 1-like protein 11 (c106023.graph_c0), which is involved in cell signal transduction and was continuously up-regulated under further cold and darkness treatment in D5 and PC. *S. trochoidea* is a worldwide species and is able to proliferate in a broad range of temperatures (5–30 ℃) [29]. Indeed, only a small proportion of vegetative cells lost swimming ability within 2 h; this differs from the situation in *Lingulodinium polyedrum*, in which all cells cease to swim, and encystment occurs within 2–3 h of cold treatment [16]. Additionally, cold and darkness treatment was initiated 1 h before the onset of the dark period to eliminate variations due to circadian rhythms. Moreover, plant cells sense cold stress through signal perception and transduction [42], and the identification of a DEG related to signal transduction suggests possible roles of signal transduction in the formation of pellicle cysts induced by cold and darkness (see below). Gene expression patterns obviously differed at 5 h and in pellicle cysts, and 3,870 DEGs (3.86 % of total unigenes) were obtained between the pellicle cysts and vegetative cells of *S. trochoidea*, which is greater than the number recorded in the dinoflagellate *L. polyedrum* (0.18 %) [16].

### Signal transduction and regulation

**Fig. 3:**
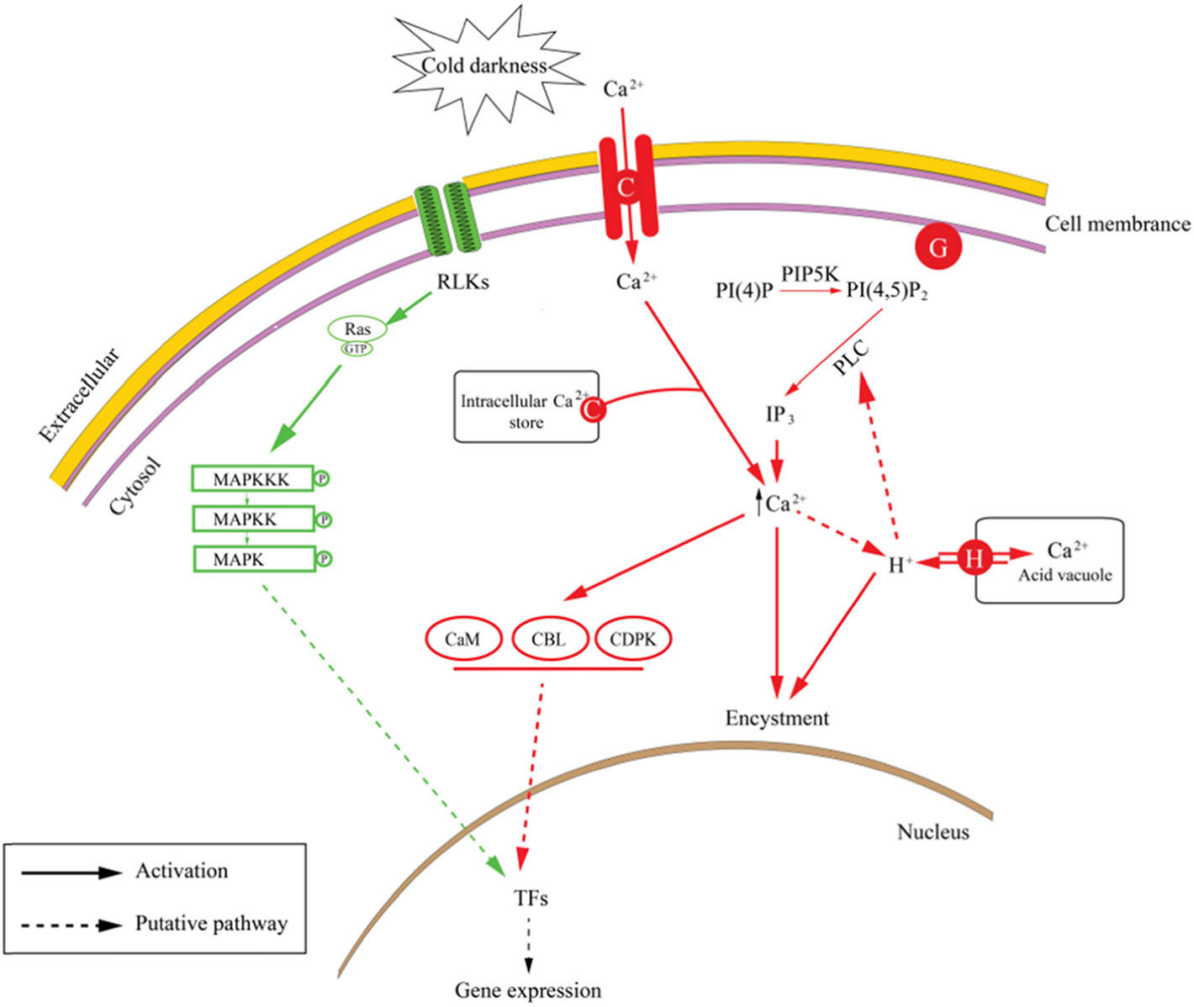
The signal transduction processes during encystment of *Scrippsiella trochoidea* induced by cold and darkness. Red lines and green lines represent Ca^2+^-dependent signaling process and MAPK signaling process, respectively. Solid arrows and dotted arrows represent positive regulation and putative pathway, respectively. RLKs: receptor like kinases; CaM: calmodulin; CBL: calcineurin B-like; CDPK: Ca^2+^-dependent protein kinase; PI(4)P: phosphatidylinositol 4-phosphate; PIP5K: phosphatidylinositol 4-phosphate 5-kinases; PI(4,5)P_2_: phosphatidylinositol 4,5-bisphosphate; PLC: phospholipase C; IP_3_: inositol 1,4,5-trisphosphate; TFs: transcription factors; G: G protein; P: phosphorylation; C: calcium-transporting ATPase; H: V-type H^+^ ATPase. This figure was modified from figures in Tsim et al. (1997) and Wu et al. (2016)

Our transcriptome data clearly showed that numerous DEGs related to Ca^2+^-dependent signaling, phospholipid signaling and protein kinases were involved in signal perception and transduction during the formation of pellicle cysts. To further link signal recognition and transduction with transcriptional regulation, we constructed a signal transduction model based on previous studies [43–45] and our transcriptome data (Fig. 3). Multiple lines of evidence have established that a rapid increase in cytosolic calcium mediated by membrane rigidification-activated mechano-sensitive or ligand-activated Ca^2+^ channels is one of the major signaling events triggered by cold stress [42, 45, 46]. Here, we identified several up-regulated genes responsible for calcium transport, including 4 genes encoding plasma membrane type calcium-transporting ATPase (PMCA, c93582.graph_c0) and endoplasmic reticulum type calcium-transporting ATPase (ERCA, c99259.graph_c2, c29254.graph_c0, c98508.graph_c1) as well as multiple genes encoding calcium channel proteins. In addition, receptor-induced elevation of cytosolic Ca^2+^ is often mediated by G protein-regulated phospholipase C (PLC) [43]. Our results identified 2 G proteins (~ 2.4-fold), 2 GTP-binding proteins (1 up-regulated and 1 down-regulated) and one phospholipase C2-like protein (2.6-fold) as well as PIP5Ks (2 up-regulated and 1 down-regulated) that catalyse phosphorylation at the D-5 position of the inositol ring of phosphatidylinositol 4-phosphate (PI(4)P), the key regulatory enzyme responsible for PI(4,5)P_2_ synthesis [47]. Moreover, it has been reported that intracellular acidification plays a vital role in the encystment of dinoflagellates. In this process, IP_3_-induced Ca^2+^ mobilization triggers proton release from the acid vacuole via a V-type H^+^ ATPase, intracellular acidification may in turn activate PLC [48, 49]. Several case studies have demonstrated that the activation of genes encoding PMCA, PIP5K, PLC and the increase in intracellular Ca^2+^ may trigger encystment of ciliates and dinoflagellates [43, 50, 51]. Consistent with these findings, in the present study, genes involved in Ca^2+^ transport were up-regulated, suggesting that elevation of intracellular Ca^2+^ may be a common mechanism leading to encystment in cyst-forming species. The subsequent regulation of gene transcription may be mediated by other Ca^2+^-dependent signaling molecules. Accordingly, we captured a total of 23 DEGs encoding CaMs, CBLs and CDPK family proteins, these proteins perceive intracellular changes in Ca^2+^ levels and translate them into specific phosphorylation events to initiate downstream signaling processes [45, 52, 53]. Additionally, we observed one gene encoding MAPKKK3 (c24727.graph_c0) that exhibited 2.9-fold induction. The MAPK pathway could thus be activated by MAPKKK3 phosphorylation. This change was associated with the activation of both ras-domain containing proteins (c65055.graph_c1, c84239.graph_c0) and RLK (c104049.graph_c0) by connecting the MAPK cascades to external signals [54] (Fig. 3). On the other hand, Deng et al. [40] demonstrated a vital role of abscisic acid (ABA) in regulating resting cyst formation and dormancy of *S*. *trochoidea*. However, in our data, the expressions of 11 genes related to ABA biosynthesis (*ZEP*, *NCED*, *AAO*) and catabolism (*ABAH*) were unchanged, suggesting ABA may not play a role in the formation of pellicle cysts of *S*. *trochoidea* induced by cold and darkness.

Plants under various stresses, including low temperature, show the activation of multiple transcription factors (TFs), which in turn regulate the global gene expression pattern [16, 55, 56]. As mentioned above, a variety of signaling pathway components were triggered during encystment, including MAPK cascades, Ca^2+^ and phospholipid second messengers, all of which induce the activation of TFs and promote/repress the expression of cold-responsive genes [44, 45, 53]. In our data, several TFs (e.g. MYB, bZIP, HSP, NAC) and cold-responsive genes (e.g. *P5CS*, *LEA*, *RCI2A*, *FAD*) were differentially expressed. However, TFs and their target genes in dinoflagellates are poorly understood, and further investigations are needed to obtain a more comprehensive view in this context and elucidate the underlying links between TFs and encystment. Taken together, the findings associated with the cold and darkness induced encystment of *S. trochoidea* obtained in our study suggest a signal mechanism by which dinoflagellates can integrate multiple pathways to transduce external signals to the nucleus and subsequently regulate gene expression.

### Energy homeostasis

Energy production is an intrinsic feature that is critical for the cellular homeostasis of cold stress responses, and plants employ a combination of mechanisms to balance energy homeostasis [57, 58]. Here, we combined transcriptome and metabolome data to construct a gene-to-metabolite network to achieve a more comprehensive understanding of energy metabolism during encystment. Glycolysis or oxidation of carbohydrates can generate a small amount of energy with pyruvate as the final product. Pyruvate can subsequently be completely oxidized to CO_2_ for the efficient production of ATP via TCA cycle and oxidative phosphorylation. Glucose is a precursor in glycolysis, and the dramatic accumulation of glucose was highly facilitated by genes related to glycan hydrolysis (9 DEGs) that presented more than 2.2-fold up-regulation during the formation of pellicle cysts. A similar observation was previously recorded in *S. trochoidea* resting cysts, in which glucose was shown to be the dominant sugar component and presented notably higher levels than in vegetative cells [59]. However, opposite transcription patterns of other genes encoding enzymes involved in the subsequent steps of glycolysis as well as TCA cycle and oxidative phosphorylation were observed at 5 h and in pellicle cysts. At 5 h, 19 genes associated with glycolysis, TCA cycle and oxidative phosphorylation were up-regulated; these genes encoding proteins such as pyruvate dehydrogenase, citrate synthase and ATP synthase subunit beta, which are the key regulatory enzymes of these pathways. In pellicle cysts, 10 of these DEGs were down-regulated by 2.0-3.8-fold. In addition, two genes encoding ADK (c83286.graph_c0, c115293.graph_c0), which plays a crucial role in regulating the right equilibrium between ADP and ATP levels, presented ~ 2.4-fold up-regulation at 5 h, and one of them (c83286.graph_c0) was down-regulated by 2.9-fold in pellicle cysts. Accordingly, the respiration rate was found to initially increase and then decrease to almost undetectable levels during the encystment of the dinoflagellate *Scrippsiella hangoei* [17]. These results suggest that the transformation from vegetative cells into pellicle cysts initially (5 h) increases ATP production to cope with cold and darkness stress, but after transformation into pellicle cysts, the metabolic activity is sufficiently reduced.

Mitochondrial β-oxidation is the major pathway by which fatty acids (FAs) are oxidized to yield energy. At 5 h, a reduction in oleic acid as well as an increased glycerol 3-phosphate, with a concomitant increase in the expression of genes involved in FA oxidation suggested that β-oxidation was enhanced. Cold and darkness treatment disrupted the photosynthetic capability of *S*. *trochoidea*, as evidenced by the decreased expression of 16 photosynthetic genes, including 2 genes encoding ribulose 1, 5-bisphosphate carboxylase, the key enzyme responsible for carbon fixation. The reduction in photosynthesis and the burst of FA β-oxidation probably indicated that the cells became carbon limited. In pellicle cysts, 2 DEGs encoding acyl-CoA dehydrogenase were up-regulated, whereas 3 DEGs involved in the next steps of β-oxidation showed greatly decreased expression. We also found that acetyl-CoA carboxylase (ACC, c72766.graph_c0), which catalyses the first committed step of FA biosynthesis, was up-regulated in pellicle cysts. ACC catalyses the production of malonyl-CoA, which is known to inhibit FA β-oxidation. Together, these results suggested that β-oxidation was active at 5 h to meet energy demands and/or increase the availability of reduced NADH and FADH_2_ for oxidative stress protection, whereas in pellicle cysts, this process became inactive. Intriguingly, 2 FA desaturases (c67979.graph_c0, c95737.graph_c0) were induced at 5 h but repressed in pellicle cysts. The down-regulation of FA desaturases contradicted with the accumulation of PUFAs (e.g. linolenic acid, arachidonic acid and palmitoleic acid). As desaturases are slow enzymes, hence PUFAs may accumulate after a certain time (e.g. in pellicle cysts) [60].

Several proteinogenic amino acids were increased during the formation of pellicle cysts. The accumulation and synthesis of amino acids are common responses to various stresses, including low temperature [61, 62]. Amino acids may be produced as an alternative energy source in plants [63]. However, the signal levels of transcripts involved in amino acid biosynthesis (which increased, remained unchanged or even decreased) are inconsistent with amino acid accumulation indicating that metabolic adjustments emanate from both transcript abundance and regulatory processes independent of transcript abundance [61, 64]. Glutamate and glutamine-associated pathways are important for the metabolism of nitrogen, in our data, the accumulation of glutamate and glutamine in pellicle cysts occurred even though ferredoxin-dependent glutamate synthase (*Fd-GOGAT*) and glutamine synthetase (*GS*) transcripts remained unaltered. The apparent contradiction of the accumulation of glutamate and glutamine concomitant with the down-regulation of *Fd-GOGAT* and *GS* has been reported in *Arabidopsis thaliana* [61]. Similarly, glutamate is the dominant amino acid in *S. trochoidea* resting cysts [59]. Among proteinogenic amino acids, proline has attracted much attention because it robustly accumulates under stress conditions [65, 66], and a correlation between proline accumulation and the acquisition of stress tolerance has been verified [67]. A dramatic increase in proline was observed in pellicle cysts, with a concomitant elevation of the expression of *P5CS* (c103423.graph_c0, 4.1-fold) and repression of the expression of *PDH* (c87955.graph_c0, 6.0-fold), which are the rate-limiting enzymes in proline biosynthesis and degradation, respectively. We observed a 5.2-fold increase in the abundance of arginine with concomitant decreases in the abundance of citrulline and ornithine (0.46-fold) as well as reduced expression of the urea transporter (c113201.graph_c0, 3.1-fold), indicating that arginine catabolism was repressed in pellicle cysts. Lirdwitayaprasit et al. [59] showed that arginine content was notably higher in cysts than in all stages of vegetative cells and proposed that nitrogen may be stored in cysts partly as arginine, which is then used as a source of nitrogen during germination. Finally, the newly formed cyst wall may prevent oxygen exchange in pellicle cysts, which may provide a possible explanation for the notably increased alanine concentrations (2.8-fold) because alanine is the major end-product in the anaerobic breakdown of proteins, and its presence is an early indicator of acute anaerobiosis [62].

Not only is the metabolic reprogramming of carbohydrates and amino acids critical for energy generation or storage but also these metabolites are recognized osmolytes that share compatible solute-like properties. These metabolites that accumulated in response to cold and darkness included several sugars (glucose, fructose and trehalose), multiple PUFAs (arachidonic acid and palmitoleic acid), amino acids (proline, glycine, glutamate) and various nitrogen-containing compounds, such as betaine and GABA, which are characterized by a common occurrence in higher plants and marine planktonic organisms under various stresses and function as osmoprotectants and ROS scavengers to stabilize or protect proteins and membranes [44, 68, 69].

### Stress response

It is now well established that encystment is a physiological response to biotic and abiotic stress, and pellicle cysts are thought to endure poor environmental conditions such as low temperature, darkness and parasite attacks. At 5 h, 4 genes encoding HSPs were up-regulated, while in pellicle cysts, the majority of *HSPs* (13 out of 15) as well as multiple chaperone proteins were down-regulated. HSPs, which are most notably known for their role in responding to environmental cues, also exhibit housekeeping functions by acting as chaperones during protein folding [70, 71]. With the marked increase in gene expression observed at 5 h, HSPs were needed to perform chaperone functions. However, due to the highly reduced metabolism in cysts, the response of *HSPs* may indicate a decreased need for chaperone functions. Intriguingly, 2 genes annotated as *CSPs* were elevated at 5 h but showed no change in pellicle cysts. CSPs are a group of multifunctional RNA binding proteins that are commonly induced upon cold shock [72]. However, they also play a role in many cellular processes under normal growth conditions [73]. Previous research showed that the abundance of CSPs remained unchanged in temporary cysts of *L. polyedrum* and suggested that CSPs do not function in cold adaptation in dinoflagellates but instead act as regulators of gene expression [16]. Our results seemed to support this view.

Various abiotic stresses lead to the overproduction of ROS in plants, which cause damage to proteins, lipids, carbohydrates and DNA, ultimately resulting in oxidative stress [74]. Plants possess efficient enzymatic antioxidant defence systems to protect their cells from oxidative damage by scavenging ROS [75]. Genes encoding ROS-scavenging enzymes were observed to be significantly up-regulated in *S. trochoidea* resting cysts [40]. Our data also indicated that genes encoding enzymes involved in critical pathways related to oxidative stress were up-regulated, suggesting that pellicle cysts respond to increased oxidative stress and that there is an elevated need for antioxidant systems in pellicle cysts. In addition, CYP450 catalytic cycle results in continuous production of ROS, which in turn down-regulates *CYP450* expression levels through a variety of feedback mechanisms [76]. These mechanisms provide an explanation for the dramatically reduced expression of *CYP450*.

Decreased transcript abundance of 16 DEGs associated with DNA replication, repair and chromatin assembly was observed in pellicle cysts. Six of these DEGs were annotated as poly (ADP-ribose) polymerases, a type of protein that is mainly involved in DNA repair and programmed cell death [71]. Others were described as ATP-dependent DNA helicase, DNA mismatch/damage repair protein and histone H3. These results indicate that DNA replication probably ceases in pellicle cysts and confirm the view that cell division is suspended in dinoflagellate cysts [40].

### Cell cycle

Generally, the eukaryotic cell cycle regulation is controlled at multiple points by a conserved set of proteins. The CDKs and the cyclins, that can form functional kinase complexes, in which the CDKs and cyclins act as catalytic and regulatory subunits, respectively [77]. Dinoflagellates have also been reported to exhibit the typical eukaryotic cell cycle regulation mechanisms [78]. We identified 2 *CDKs* (c44162.graph_c, c46114.graph_c2) that presented decreased expression (3.3 and 3.9-fold) in pellicle cysts. One of them (c44162.graph_c) is homologous to F-type *CDK*, which is a CDK-activating kinase (CAK) that activates A-type CDKs and functions without any binding partner [79]. Accordingly, A-type *CDK* (c46114.graph_c2), which is active during both G1-S and G2-M transitions [80], exhibited decreased expression. The transcriptional expression of 2 *cyclins* (*cyclin B* and *cyclin U*) was also repressed in pellicle cysts. *Cyclin B* (c101407.graph_c0, c103194.graph_c0) functions in mitosis, and the expression profile of *cyclin B* resembles that of *CDKB*, with elevated levels of mRNA appearing during G_2_-M transition [79]. *Cyclin U* (c84613.graph_c1) was preliminarily documented as playing a key role in mediating brassinosteroid-regulated cell division to control leaf erectness in *Oryza sativa* [81]. Finally, a gene (c45848.graph_c1) homologous to a cell division cycle protein was also shown to be repressed in pellicle cysts. Our results suggest that genes pertinent to cell cycle may be associated with the encystment of *S. trochoidea*. Since cell cycle genes appeared to be post-transcriptionally regulated in dinoflagellates [82], further proteomic analysis will be useful to reveal the underlying links between cell cycle and encystment [40].

Pellicle cysts are traditionally considered to be produced by asexual processes. It has been previously proven that pellicle cysts can be either asexual or sexual in the species *L. polyedrum* [83] and *Alexandrium taylori* [84], and *Pfiesteria* and *cryptoperidiniopsoids* [85] generate temporary cysts as a product of the sexual cycle. Our data revealed 11 DEGs (10 up-regulated and 1 down-regulated) that were potentially involved in sexual reproduction. These genes were also detected in the transcriptomes and genomes of dinoflagellate *Symbiodinium* species [86, 87], in addition to being previously reported in *S. trochoidea* [40]. Bioinformatic researches have revealed that *MEI2*-like genes are widespread in the plant kingdom [88, 89]. They are the master regulators of meiosis and encode RNA binding proteins required for premeiotic DNA synthesis as well as entry into meiosis I in the yeasts *Schizosaccharomyces pombe* and *Saccharomyces cerevisiae* and the plant *Arabidopsis* [90]. In addition, *SMC3* encodes the cohesion complex, which is required to hold sister chromatids together in diatoms [91]. Therefore, *MEI2*-like genes and *SMC3* were presumed to be involved in the sexual reproduction and encystment of *S. trochoidea*. However, in this study, it remained unclear whether the pellicle cysts of *S. trochoidea* (or part) were produced via sexual reproduction. This uncertainty makes it necessary to the ascertain ploidy levels of cysts, and further work involving approaches such as flow cytometry for determining the ploidy levels of pellicle cysts is in progress.

## Conclusions

In summary, we adopted RNA-seq and UHPLC-QTOF-MS approaches to analyse the overall transcriptome and metabolome changes during cold and darkness induced encystment of *S. trochoidea*. Consequently, massive transcriptome and metabolome reprogramming occurred during encystment. The gene-to-metabolite network demonstrated that the initial transformation from vegetative cells into pellicle cysts was a highly energy demanding process. However, the metabolism was highly reduced, and various carbohydrates and amino acids accumulated in pellicle cysts. Furthermore, dinoflagellates can integrate multiple signal transduction pathways to transduce external signals to the nucleus, leading to encystment. DEGs with roles in oxidative stress, DNA replication, cell cycle and sexual reproduction were also observed during encystment. Our results provide insights into the mechanisms underlying life cycle regulation and will facilitate investigations of the dynamics of dinoflagellate blooms.

## Methods

### Cell culture and sample preparation

The culture of *S. trochoidea* was germinated from a single *S. trochoidea* cyst that was sampled from the South China Sea and was identified using a partial 28 S rDNA sequence. The newly generated partial LSU sequence was 912 bp in length. BLAST analysis showed that the sequence was 99 % identical to the LSU of *S. trochoidea* strain BDH (GenBank KR336540). Cultures were grown in f/2-Si medium [92] prepared from 0.22 μm filtered, autoclaved seawater (salinity 29–31) at 20 ± 1℃ in a light incubator with a 12:12 h L/D cycle and a photon flux density of 100 µE m^− 2^ s^− 1^. Penicillin and streptomycin were added to the medium before inoculation (final concentration, 10 µg mL^− 1^) to inhibit bacterial growth.

Cultures in the exponential phase were inoculated into 500 mL flasks containing 350 mL medium to achieve an approximate cell density of 2.5 × 10^4^ cells mL^− 1^ (n = 6) and were maintained in a refrigerator at 8 ± 1 ℃ for 3 days to induce pellicle cyst formation. Samples were collected at 2 h, 5 h, and day 3. Vegetative cells in the exponential phase before the experiment were harvested as a control (CK). Cells were checked under a light microscope (Nikon ECLIPSE TS2, Japan) and were harvested after 2 h (D2) and 5 h (D5) of treatment by centrifugation (6500 rpm for 6 min, Beckman Allegra 64R, USA), after which they were frozen in liquid nitrogen and stored at -80℃ until RNA and metabolite extraction. The remaining cultures were maintained with continued treatment for pellicle cyst production. At day 3, pellicle cysts (PC) were collected by centrifugation as described above. After resuspension in sterile seawater, the samples were layered on top of a 60 % (v/v) solution of Percoll (GE healthcare, Sweden) in seawater and centrifuged again (2000 rpm for 20 min). Debris settled to the bottom of the tube, while pellicle cysts remained at the interface. Pellicle cysts were collected and washed several times with sterile seawater, frozen in liquid nitrogen and stored at -80℃. Additionally, the cold and darkness treatment was initiated 1 h before the onset of the dark period. For transcriptomic and metabolomic analyses, three and six independent biological replicates were performed, respectively.

### RNA extraction, library construction and sequencing

Total RNA from each sample was extracted using TRIzol (Invitrogen, USA) according to the manufacturer’s instructions and purified using RNase-free DNase I (Qiagen, Germany) to avoid genomic DNA contamination. The quantity and quality of the total RNA were analysed via 1 % agarose gel electrophoresis and on a NanoDrop 2000 spectrophotometer (Thermo Fisher Scientific, USA). RNA integrity number (RIN) values were determined using an Agilent 2100 Bioanalyzer (Agilent Technologies, USA), and samples with RIN values ≥ 7 were used for further processes.

A total of 1 µg of RNA per sample was used for RNA sample preparation. The sequencing libraries were generated using the NEBNext Ultra RNA Library Prep Kit for Illumina (NEB, USA) following the manufacturer’s recommendations. Poly (A)-mRNA was purified from total RNA using poly-T oligo-attached magnetic beads. First strand cDNA was synthesized using random hexamer primers and M-MuLV reverse transcriptase, and second strand cDNA synthesis was subsequently performed using DNA Polymerase I and RNase H. After the adenylation of the 3^ʹ^ ends of the DNA fragments, NEBNext adaptors with a hairpin loop structure were ligated in preparation for hybridization. The library fragments were purified with the AMPure XP system (Beckman Coulter, Beverly, USA) to preferentially select cDNA fragments of 240 bp in length. Then, the USER Enzyme (NEB, USA) was applied to size-selected, adaptor-ligated cDNA, and PCR was performed with Phusion High-Fidelity DNA polymerase, universal PCR primers and an Index (X) primer. The PCR products were purified using the AMPure XP system, and the quality of the library was assessed on the Agilent Bioanalyzer 2100 system. Finally, the clustering of the index-coded samples was performed on a cBot Cluster Generation System using TruSeq PE Cluster Kit v3-cBot-HS (Illumina) according to the manufacturer’s instructions. After cluster generation, the library preparations were sequenced on the Illumina HiSeq 2000 platform to generate paired-end reads by the Biomarker Biotechnology Corporation (Beijing, China). The libraries from each biological replicate yielded at least 6 GB of raw data. The raw reads of transcriptome were deposited in the NCBI Sequence Read Archive (SRA) with the accession numbers SRR14868303, SRR14868302, SRR14868301 and SRR14868300 corresponding to the CK, D2, D5 and PC group, respectively.

### ***De novo*** transcriptome assembly and functional annotation

Raw data in FASTQ format were first processed with in-house Perl scripts (Biomarker Biotechnology Corporation, Beijing, China). In this step, raw reads were firstly preprocessed by removing reads containing adaptors using cutadapter (v1.9.1), ploy-N > 10 % and low quality reads with quality scores less than Q30 (85 %) from the raw data. The Q30 values, GC contents and sequence duplication levels of the clean data were calculated. The high quality reads obtained after the above series of quality controls were referred to as clean reads and stored in FASTQ format. All downstream analyses were based on clean reads. Due to the absence of genomic information, *de novo* assembly was performed using the Trinity platform with the parameters “K-mer = 25, group_pairs_distance = 500, min_glue = 2, min_kmer_cov = 2” [93]. Subsequently, the TGICL software system was used to cluster the potential unigenes and generate a single set of non-redundant unigenes [94]. Furthermore, clean reads were mapped to the assembled unigenes to estimate the efficiency of short-read usage during *de novo* assembly.

Functional annotation of all unigenes was carried out by searches against public databases: Nr (non-redundant protein sequence database, ftp://ftp.ncbi.nih.gov/blast/db/), Swiss-Prot (a manually annotated and non-redundant protein sequence database, http://www.uniprot.org/), GO (Gene Ontology, http://www.geneontology.org/), KOG (the database of Clusters of Protein homology, http://www.ncbi.nlm.nih.gov/KOG/) and KEGG (Kyoto Encyclopedia of Genes and Genomes, http://www.genome.jp/kegg/) using BLAST (v2.1.31, http://blast.ncbi.nlm.nih.gov/Blast.cgi) with a cut-off E-value of 10^− 5^. Furthermore, unigenes with poor BLAST hit descriptions or those that lacked a database match were translated into potential amino acid sequences using ORFpredictor [95]. Then, translated potential proteins were annotated using HMMER software (v3.1b2, http://hmmer.org/) against Pfam (homologous protein family database, http://pfam.xfam.org/) (E-value ≤ 1e^− 10^).

### Quantification of gene expression and differential expression analysis

For gene expression analysis, clean reads were mapped back onto the assembled transcriptome to obtain count values of unigenes using Bowtie [96], and then normalized into fragments per kilobase of transcript per million mapped reads (FPKM) values using RESM [97]. Meanwhile, the Pearson correlation coefficient between three independent replicates was calculated for verifying the gene expression profiles. The expression values between the same treatments were highly correlated (r^2^ > 0.98), indicating that all collected samples were well processed (supplementary Fig. S5). Differential expression analysis was performed by modeling count values with negative binomial distributions described in the DESeq2 [98], and genes with false discovery rate (FDR) < 0.01 and absolute log_2_ fold-change (log_2_FC) ≥ 1 were assigned as differentially expressed genes (DEGs).

For functional enrichment analysis of DEGs, Blast2GO software was used to assign GO terms describing biological processes, molecular function, and cellular component [99], and the R package topGO was employed to evaluate the significance of enrichment using the default algorithm and Kolmogorov-Smirnov (KS) test (*p*-value ≤ 0.01) [100]. Furthermore, all DEGs were mapped to the KEGG database and subjected to searches for significantly enriched metabolic pathways using KOBAS2.0 with a Bonferroni-adjusted *p*-value ≤ 0.01 [101].

### Metabolite profiling

Samples (approximately 10^6^ cells or cysts per sample) were isolated in 1 mL of an extract solution (acetonitrile:methanol:water = 2:2:1) containing an internal standard (L-2-chlorophenylalanine, 2 µg mL^− 1^). The samples were homogenized at 35 Hz for 4 min and then sonicated for 5 min in an ice-water bath, and the cycle was repeated 2 times. After incubation at -40 ℃ for 1 h, the samples were centrifuged at 10,000 rpm for 15 min at 4 ℃ (Thermo Fisher Scientific Heraeus Fresco17, USA), and 250 µL of the supernatant was transferred to a fresh tube and dried in a vacuum concentrator at 37 ℃. The dried samples were reconstituted in 300 µL of 50 % acetonitrile by sonication on ice for 10 min and centrifuged at 13,000 rpm for 15 min at 4 ℃. A 75 µL aliquot of the supernatant was then transferred to a fresh glass vial for LC/MS analysis. Quality control (QC) samples were prepared by mixing an equal aliquot of the supernatants from all of the samples.

The UHPLC separation was carried out using a 1290 Infinity series UHPLC System (Agilent Technologies, USA) equipped with a UPLC BEH Amide column (2.1*100 mm, 1.7 μm, Waters). The mobile phase consisted of 25 mM ammonium acetate and 25 mM ammonia hydroxide in water (pH = 9.75) (A) and acetonitrile (B). The analysis was carried out with the following elution gradient: 0 ~ 0.5 min, 5 % A, 95 % B; 0.5 ~ 7.0 min, 5 %~35 % A, 95 %~65 % B; 7.0 ~ 8.0 min, 35 %~60 % A, 65 %~40 % B; 8.0 ~ 9.0 min, 60 % A, 40 % B; 9.0 ~ 9.1 min, 60 %~5 % A, 40 %~95 % B; 9.1 ~ 12.0 min, 5 % A, 95 % B. The column temperature was 25 ℃. The autosampler temperature was 4 ℃, and the injection volume was 1 µL. A TripleTOF 6600 mass spectrometer (AB Sciex) was used to acquire MS/MS spectra on an information-dependent basis (IDA) during an LC/MS experiment. In this mode, the acquisition software (Analyst TF 1.7, AB Sciex) continuously evaluates the full scan survey MS data as it collects and triggers the acquisition of MS/MS spectra depending on preselected criteria. In each cycle, the 12 most intense precursor ions with intensities above 100 were chosen for MS/MS analysis at a collision energy (CE) of 30 eV. The cycle time was 0.56 s. ESI source conditions were set as follows: gas 1 60 psi, gas 2 60 psi, curtain gas 35 psi, source temperature 600 ℃, declustering potential 60 V, and ion spray voltage floating (ISVF) at 5000 V or -4000 V in positive or negative mode, respectively.

The raw MS data were converted to the mzXML format with ProteoWizard and processed with the R package XCMS [102]. The process included peak deconvolution, alignment and integration, and the minfrac and cut off values were set as 0.5 and 0.6, respectively. The relative abundance of metabolites was estimated by dividing the peak area of each metabolite by the total peak area of all metabolites in the sample to minimize the influence induced by fluctuations of ion signals during the experiments. The in-house MS2 database was applied for metabolite identification using the Human Metabolome Database (HMDB) [103] and KEGG [104]. OPLS-DA and *t*-test were used to conduct statistical data analysis [105, 106]. Cross validation for OPLS-DA was performed using a permutation testing set for the selected y variable and monitoring the statistical significance of R^2^ and Q^2^ values. Differentially accumulated metabolites were defined according to the following criteria: fold change > 2, *p*-value < 0.05 and variable importance in the projection (VIP) > 1.

## Supplementary Information

**Additional file 1: Supplementary Table S1** Overview of transcriptome sequencing data of *Scrippsiella trochoidea*. **Supplementary Table S2** Summary of functional annotation of assembled unigenes.

**Additional file 2: Supplementary Figure S1** The species distribution of the result of Nr annotation. **Supplementary Figure S2** GO annotation of assembled unigenes for *Scrippsiella trochoidea*. **Supplementary Figure S3** KOG function classification of consensus sequences of assembled unigenes for *Scrippsiella trochoidea*.

**Additional file 3: Supplementary Table S3** Full lists of differentially expressed genes with annotations during encystment.

**Additional file 4: Supplementary Table S4** Significantly enriched GO terms (p < 0.01) of the differentially expressed genes.

**Additional file 5: Supplementary Table S5** KEGG pathway enrichment of differentially expressed genes.

**Additional file 6: Supplementary Table S6** Differentially expressed genes related to signal transduction during encystment.

**Additional file 7: Supplementary Table S7** Differentially expressed genes associated with energy metabolism during encystment.

**Additional file 8: Supplementary Table S8** Differentially expressed genes related to cell stress response.

**Additional file 9: Supplementary Table S9** Differentially expressed genes involved in cell cycle in pellicle cysts.

**Additional file 10: Supplementary Figure S4** Orthogonal projections to latent structures-discriminant analysis of MS data, all detected molecular features were analysed. (a) CK vs. D5 in positive ion mode; (b) CK vs. D5 in negative ion mode; (c) CK vs. PC in positive ion mode; (d) CK vs. PC in negative ion mode. CK represents the control group of vegetative cells in the exponential phase, D5 represents the group of cold and darkness treatment for five hours, PC represents the group of pellicle cysts.

**Additional file 11: Supplementary Table S10** Full lists of differentially accumulated metabolites with annotations during encystment.

**Additional file 12: Supplementary Figure S5** Correlation between biological replicates.

## Data Availability

The datasets generated during the current study were deposited in the NCBI Sequence Read Archive (SRA) with the accession numbers SRR14868303, SRR14868302, SRR14868301 and SRR14868300 corresponding to the CK, D2, D5 and PC group, respectively.
