# Supplementary material for: Transcriptome and metabolome analyses of cold and darkness-induced pellicle cysts of Scrippsiella trochoidea
Source: BMC Genomics. 2021 Jul 10;22:526. doi: 10.1186/s12864-021-07840-7 (PMC8272339; doi:10.1186/s12864-021-07840-7)
Supplement: Supplementary file 1 — Additional file 1: Supplementary Table S1 Overview of transcriptome sequencing data of Scrippsiella trochoidea. Supplementary Table S2 Summary of functional annotation of assembled unigenes. [file 12864_2021_7840_MOESM1_ESM.docx]

**Supplementary Table S1 Overview of transcriptome sequencing data of *Scrippsiella trochoidea***

| Sample | Clean reads | Clean bases | Mapped reads | Mapped ratio (%) | Q30 (%) | GC content (%) |
| --- | --- | --- | --- | --- | --- | --- |
| CK1 | 32,235,688 | 9,598,523,006 | 25,509,304 | 79.13 | 92.86 | 61.49 |
| CK2 | 28,963,657 | 8,644,164,058 | 22,774,902 | 78.63 | 93.83 | 61.96 |
| CK3 | 25,908,609 | 7,728,791,324 | 20,471,915 | 79.02 | 93.37 | 62.06 |
| D2-1 | 23,010,203 | 6,848,711,350 | 17,911,621 | 77.84 | 93.48 | 61.88 |
| D2-2 | 46,403,845 | 13,870,918,236 | 35,224,909 | 75.91 | 92.75 | 62.89 |
| D2-3 | 32,406,026 | 9,634,366,828 | 25,382,916 | 78.33 | 93.78 | 61.73 |
| D5-1 | 21,352,900 | 6,349,141,980 | 16,230,735 | 76.01 | 93.42 | 61.74 |
| D5-2 | 20,915,776 | 6,212,040,648 | 15,659,189 | 74.87 | 93.09 | 62.57 |
| D5-3 | 21,169,196 | 6,297,088,242 | 16,127,316 | 76.18 | 93.24 | 62.05 |
| PC1 | 24,658,204 | 7,373,749,458 | 19,222,890 | 77.96 | 95.39 | 61.52 |
| PC2 | 24,204,181 | 7,238,112,748 | 18,870,068 | 77.96 | 95.40 | 61.84 |
| PC3 | 20,692,074 | 6,186,507,750 | 16,208,460 | 78.33 | 95.29 | 61.90 |
| Total | 321,920,359 | 95,982,115,628 | 249,594,225 | 77.53 | - | - |

CK refers to the control group of vegetative cells in the exponential phase, D2 and D5 refer to the groups of cold and darkness treatment for two hours and five hours, respectively, PC refers to the group of pellicle cysts. Q30 represents proportion of nucleotides with quality value larger than 30

**Supplementary Table S2 Summary of functional annotation of assembled unigenes**

| Database | Annotated number | Percentage (%) |
| --- | --- | --- |
| Nr | 43272 | 43.21 |
| GO | 8604 | 8.59 |
| COG | 16603 | 16.58 |
| KOG | 18301 | 18.28 |
| KEGG | 11364 | 11.35 |
| Swiss-Prot | 15079 | 15.06 |
| Pfam | 31883 | 31.84 |
| All annotated | 46512 | 46.45 |
