# Supplementary material for: Transcriptome and metabolome analyses of cold and darkness-induced pellicle cysts of Scrippsiella trochoidea
Source: BMC Genomics. 2021 Jul 10;22:526. doi: 10.1186/s12864-021-07840-7 (PMC8272339; doi:10.1186/s12864-021-07840-7)
Supplement: Supplementary file 2 — Additional file 2: Supplementary Figure S1 The species distribution of the result of Nr annotation. Supplementary Figure S2 GO annotation of assembled unigenes for Scrippsiella trochoidea. Supplementary Figure S3 KOG function classification of consensus sequences of assembled unigenes for Scrippsiella trochoidea. [file 12864_2021_7840_MOESM2_ESM.docx]

**
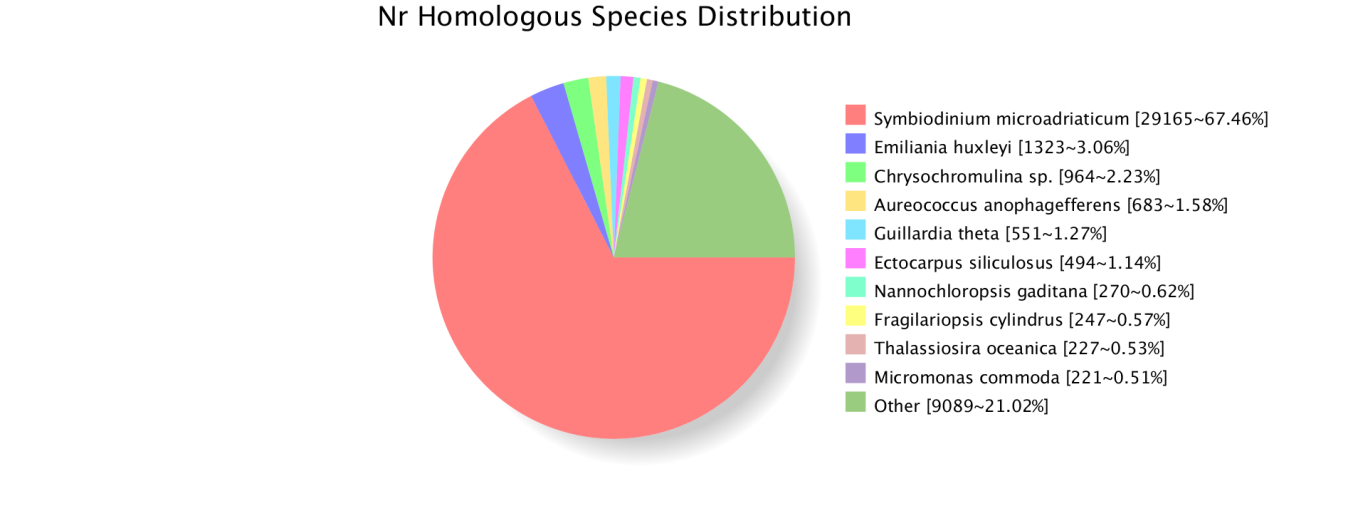
**

Supplementary Fig. S1 The species distribution of the result of Nr annotation. The Numbers in the square brackets represent the amount and percentage of unigenes annotated to corresponding species

**
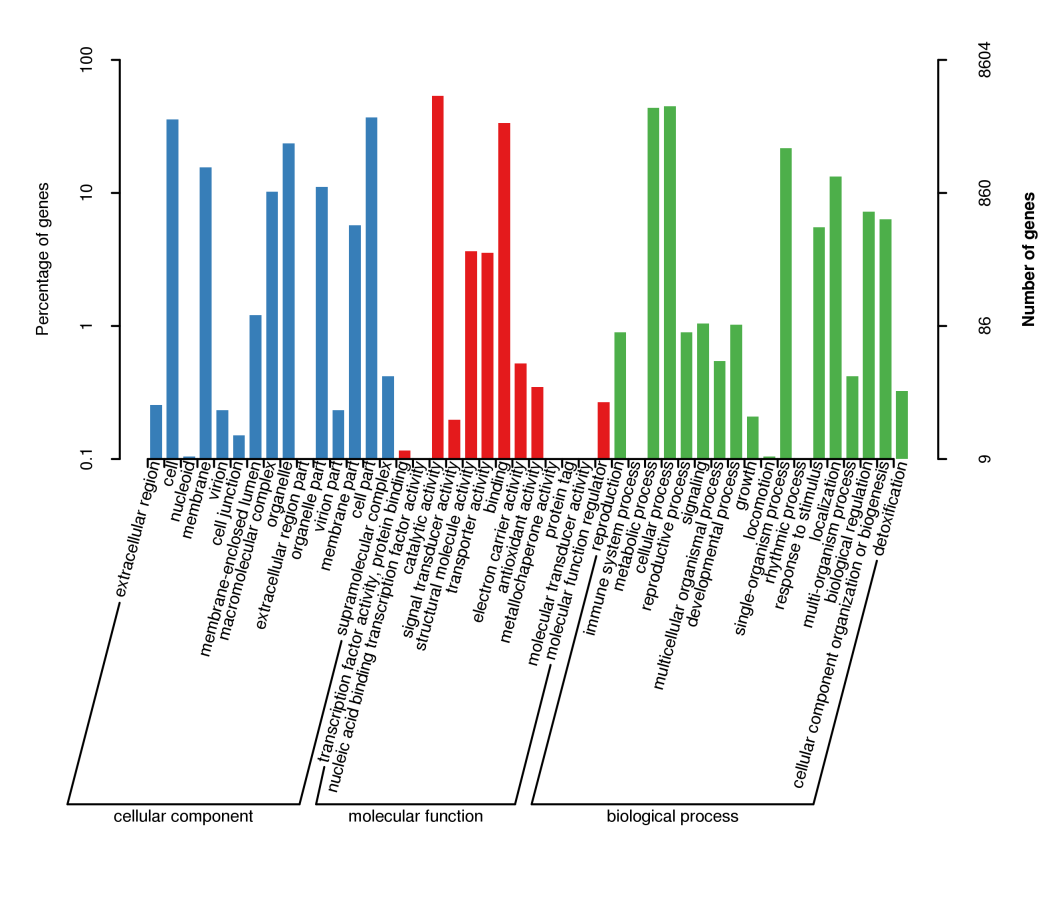
**

Supplementary Fig. S2 GO annotation of assembled unigenes for *Scrippsiella trochoidea*. X axis represents GO categories, y axis represents the number and percentage of unigenes in each GO category

**
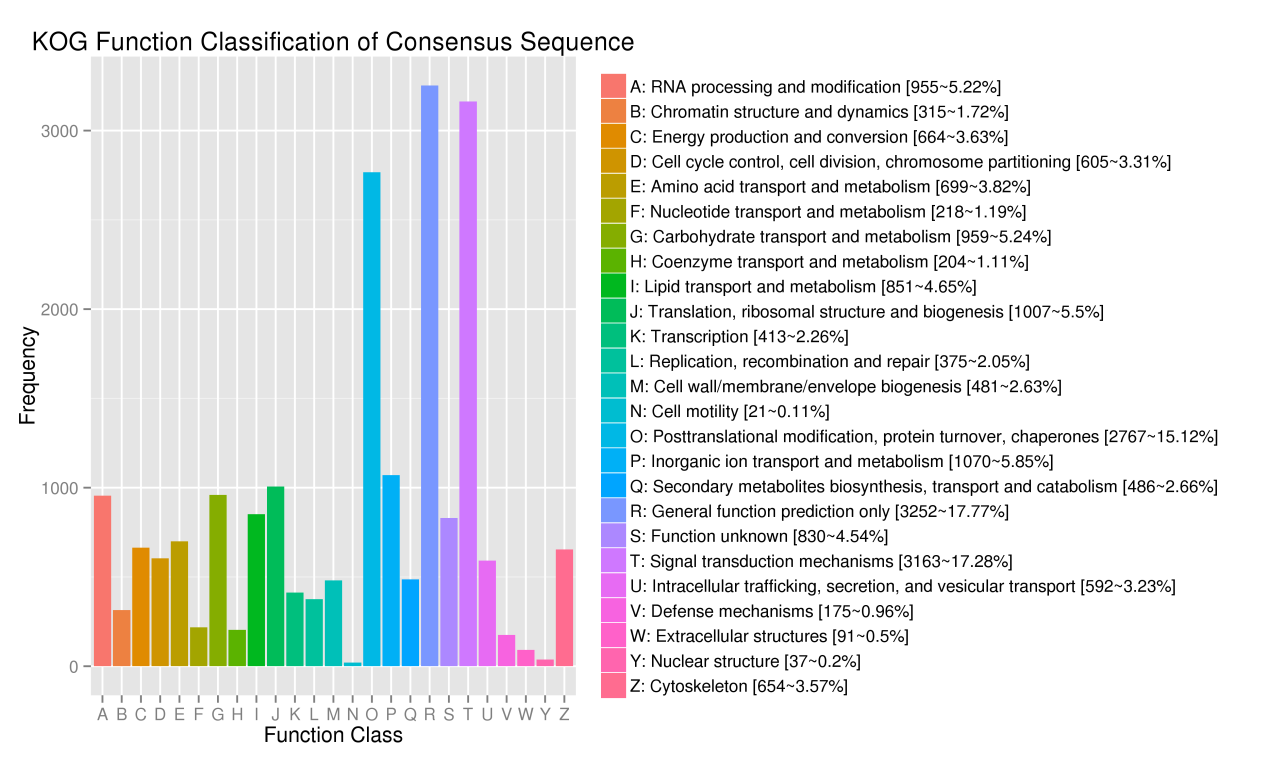
**

Supplementary Fig. S3 KOG function classification of consensus sequences of assembled unigenes for *Scrippsiella trochoidea*. X axis represents the 25 categories of KOG database, y axis represents the number of unigenes in each KOG category
