# Supplementary material for: Transcriptome and metabolome analyses of cold and darkness-induced pellicle cysts of Scrippsiella trochoidea
Source: BMC Genomics. 2021 Jul 10;22:526. doi: 10.1186/s12864-021-07840-7 (PMC8272339; doi:10.1186/s12864-021-07840-7)
Supplement: Supplementary file 10 — Additional file 10: Supplementary Figure S4 Orthogonal projections to latent structures-discriminant analysis of MS data, all detected molecular features were analysed. (a) CK vs. D5 in positive ion mode; (b) CK vs. D5 in negative ion mode; (c) CK vs. PC in positive ion mode; (d) CK vs. PC in negative ion mode. CK represents the control group of vegetative cells in the exponential phase, D5 represents the group of cold and darkness treatment for five hours, PC represents the group of pellicle cysts. [file 12864_2021_7840_MOESM10_ESM.docx]

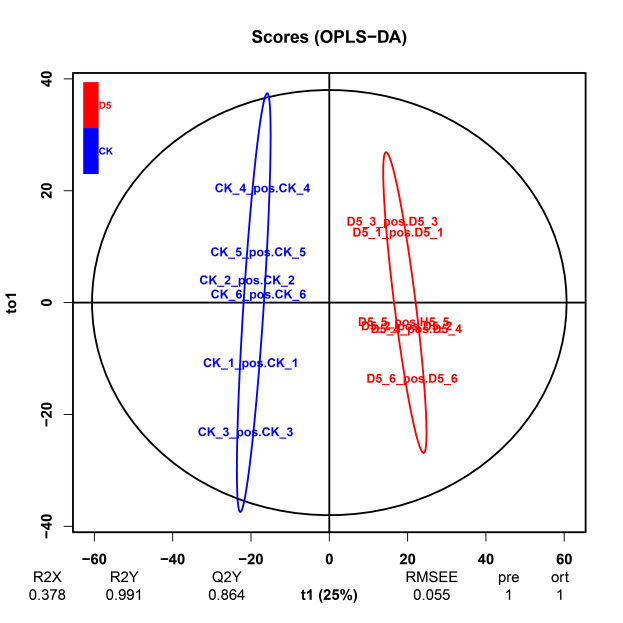

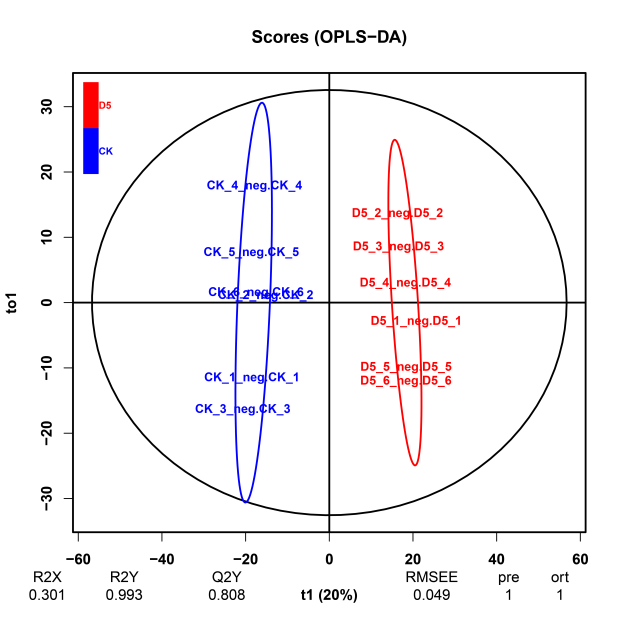


b

a


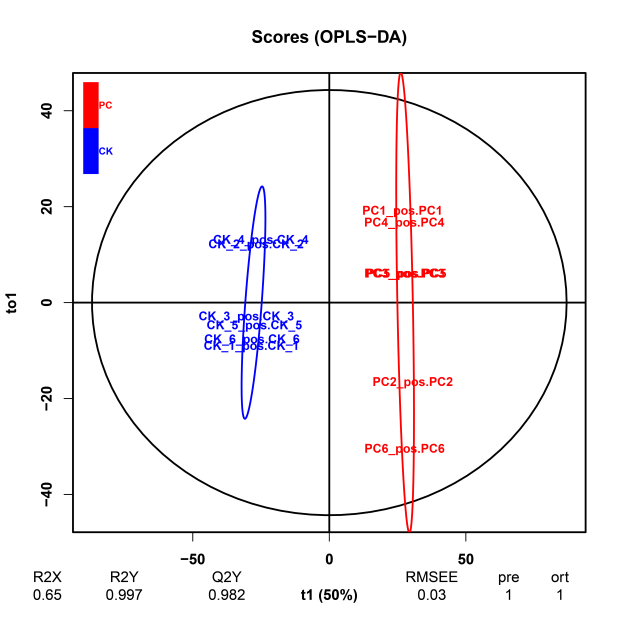

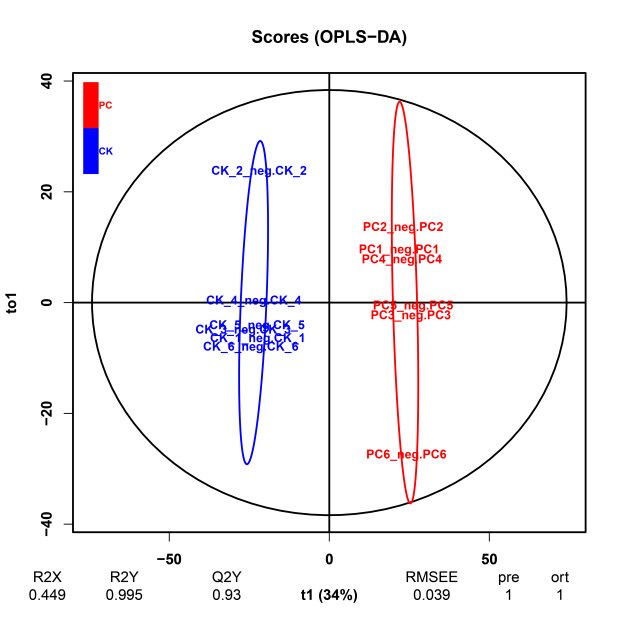


c

d

Fig. S4 Orthogonal projections to latent structures-discriminant analysis of MS data, all detected molecular features were analysed. (a) CK vs. D5 in positive ion mode; (b) CK vs. D5 in negative ion mode; (c) CK vs. PC in positive ion mode; (d) CK vs. PC in negative ion mode. CK represents the control group of vegetative cells in the exponential phase, D5 represents the group of cold and darkness treatment for five hours, PC represents the group of pellicle cysts
