# Supplementary material for: Transcriptome and metabolome analyses of cold and darkness-induced pellicle cysts of Scrippsiella trochoidea
Source: BMC Genomics. 2021 Jul 10;22:526. doi: 10.1186/s12864-021-07840-7 (PMC8272339; doi:10.1186/s12864-021-07840-7)
Supplement: Supplementary file 12 — Additional file 12: Supplementary Figure S5 Correlation between biological replicates. [file 12864_2021_7840_MOESM12_ESM.docx]

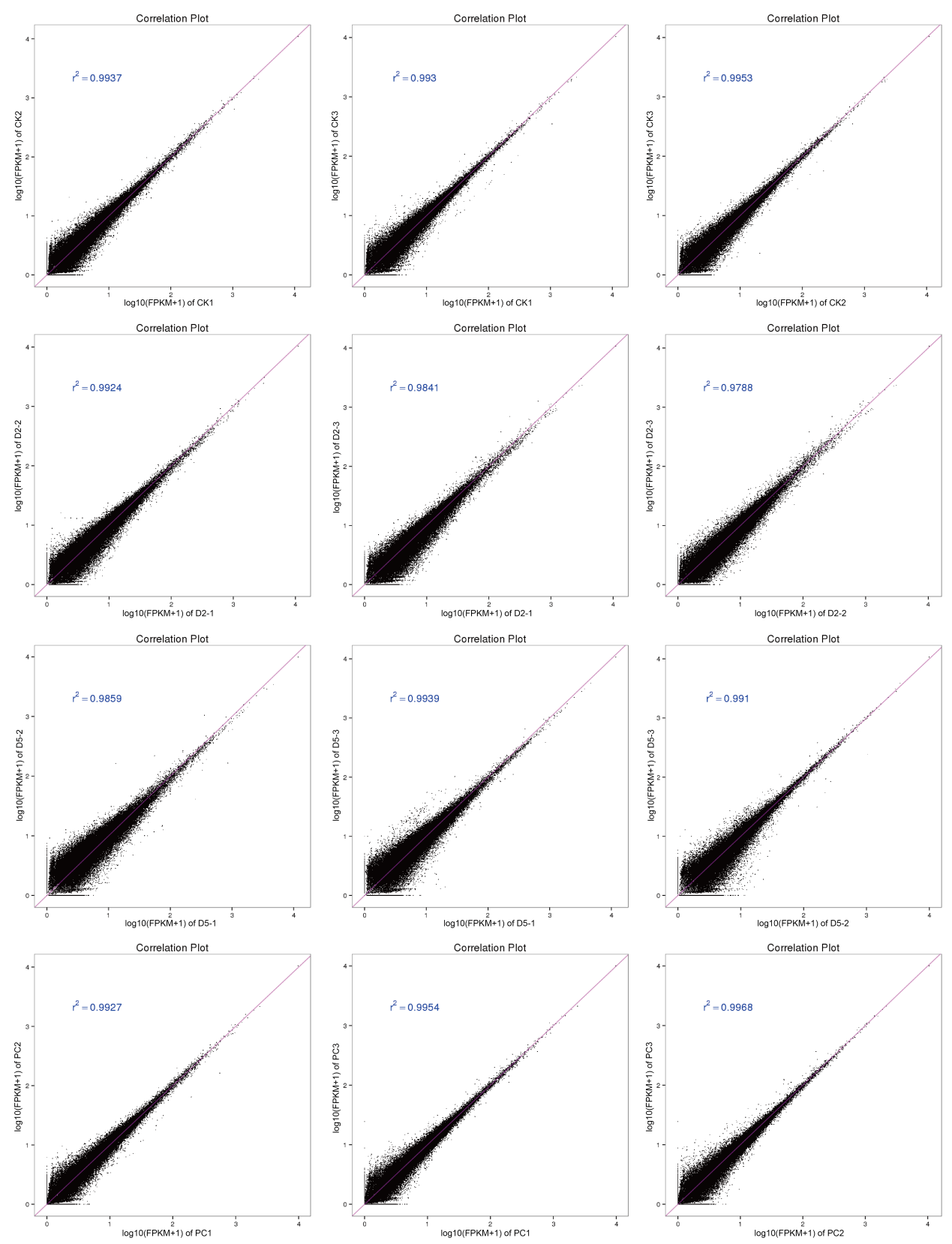


Supplementary Fig. S5 Correlation between biological replicates. For transcriptome analysis, three independent biological replicates for each treatment were used to construct RNA-seq libraries. The normalized data of log_10_ (FPKM value + 1) are highly correlated (r^2^＞0.98) between replicates in the same treatments, indicating that all collected samples were well processed
